# Detection of 2-Hydroxyglutarate by 3.0-Tesla Magnetic Resonance Spectroscopy in Gliomas with Rare IDH Mutations: Making Sense of “False-Positive” Cases

**DOI:** 10.3390/diagnostics11112129

**Published:** 2021-11-16

**Authors:** Manabu Natsumeda, Hironaka Igarashi, Ramil Gabdulkhaev, Haruhiko Takahashi, Kunio Motohashi, Ryosuke Ogura, Jun Watanabe, Yoshihiro Tsukamoto, Kouichirou Okamoto, Akiyoshi Kakita, Tsutomu Nakada, Yukihiko Fujii

**Affiliations:** 1 Department of Neurosurgery, Brain Research Institute, Niigata University, Niigata 951-8122, Japan; haruhiko.takahashi@icloud.com (H.T.); kmoto@bri.niigata-u.ac.jp (K.M.); oguryou@bri.niigata-u.ac.jp (R.O.); watanabejun1003@yahoo.co.jp (J.W.); yoshi.tsukamoto@me.com (Y.T.); yfujii@bri.niigata-u.ac.jp (Y.F.); 2 Center for Integrated Brain Sciences, Brain Research Institute, Niigata University, Niigata 951-8585, Japan; tnakada@bri.niigata-u.ac.jp; 3 Department of Pathology, Brain Research Institute, Niigata University, Niigata 951-8585, Japan; ram.gab258852@gmail.com (R.G.); kakita@bri.niigata-u.ac.jp (A.K.); 4 Department of Translational Research, Brain Research Institute, Niigata University, Niigata 951-8585, Japan; oko-okamoto@bri.niigata-u.ac.jp

**Keywords:** 2-hydroxyglutarate, glioma, magnetic resonance spectroscopy, rare IDH mutations, false-positive

## Abstract

We have previously published a study on the reliable detection of 2-hydroxyglutarate (2HG) in lower-grade gliomas by magnetic resonance spectroscopy (MRS). In this short article, we re-evaluated five glioma cases originally assessed as isocitrate dehydrogenase (IDH) wildtype, which showed a high accumulation of 2HG, and were thought to be false-positives. A new primer was used for the detection of *IDH2* mutation by Sanger sequencing. Adequate tissue for DNA analysis was available in 4 out of 5 cases. We found rare *IDH2* mutations in two cases, with *IDH2* R172W mutation in one case and *IDH2* R172K mutation in another case. Both cases had very small mutant peaks, suggesting that the tumor volume was low in the tumor samples. Thus, the specificity of MRS for detecting IDH1/2 mutations was higher (81.3%) than that originally reported (72.2%). The detection of 2HG by MRS can aid in the diagnosis of rare, non-IDH1-R132H *IDH1* and *IDH2* mutations in gliomas.

## 1. Introduction

Isocitrate dehydrogenase (IDH)-mutant gliomas produce the oncometabolite 2-hydroxyglutarate (2HG). We previously reported on the reliable detection of 2HG by 3.0-tesla magnetic resonance spectroscopy (MRS) in a cohort of 52 lower-grade glioma patients (WHO grades 2 and 3) [1]. A cutoff of 1.489 mM for 2HG yielded 100% sensitivity and a 72.2% specificity for the detection of *IDH1* or *IDH2* mutations was reported. A high level of 2HG was detected in 5 of 27 (18.5%) gliomas that were determined to be IDH-wildtype. These were thought to be false-positive results or a failure to detect rare *IDH1* or *IDH2* mutations by DNA sequencing [1]. The unambiguous detection of 2HG (chemical shift 2.25 ppm) by MRS is difficult because of a spectral overlap with glutamate (Glu; 2.43 ppm), glutamine (Gln; 2.34 ppm), and gamma-aminobutyric acid (GABA; 2.28 ppm). Recently, the tumor recurred in one of the five patients, and the patient underwent a second surgery. An analysis of *IDH1/2* mutations using Sanger sequencing revealed an *IDH2* mutation. In the current study, we re-evaluated *IDH1* and *IDH2* status in the remaining “false-positive” cases with available tissue. We found that the 2HG detection by MRS was useful in the selection of glioma cases with rare *IDH1* and *IDH2* mutations.

## 2. Materials and Methods

MRI/1H-MRS was performed using a 3.0-tesla system (Signa LX, General Electric, Waukesha, WI, USA) as previously reported [1], in accordance with the human research guidelines of the Internal Review Board of Niigata University (Approval #2017-0163) after obtaining written consent from all participants. Proton density images (Fast Spin Echo; TR/TE = 5000/40; FOV: 20 × 20 mm; matrix: 256 × 256; slice thickness: 5 mm; inter-slice gap: 2.5 mm) were captured. The slice with the largest depiction of the tumor on proton density images was selected for SVMRS. A point-resolved spectroscopic sequence (PRESS) with a chemical-shift-selective water suppression was used with the following parameters: TR: 1.5 s; TE: 30 ms; data point 512; spectral width 1000 Hz; number of acquisitions: 128–196; volume of interest (VOI): 12–20 × 12–20 × 12–20 mm). The volume of interest (VOI) was designed to minimize the suspected areas of necrosis and hemorrhage. A spectral analysis was performed using LCModel version 6.3 (Stephen Provencher, Oakville, ON, Canada) [2]. This software automatically adjusts the phase and chemical shift of the spectra, estimates the baseline, and performs eddy current corrections. Relative metabolite concentrations and their uncertainties were estimated by fitting the spectrum to a basis set of spectra acquired from individual metabolites in-solution. The basis set was provided by Dr. Steven Provencher [2] and was calibrated with an MRS phantom solution (18-cm-diameter MRS HDsphere, model 2152220; General Electric) using our MR system. Nineteen metabolites were included in the LCModel basis set, including alanine, aspartate, creatine (Cr), phosphocreatine (PCr), GABA, glucose, Gln, Glu, glycerophosphocholine (GPC), phosphocholine (PC), glutathione (GSH), 2HG, myo-inositol (Ins), lactate, N-acetylaspartate (NAA), N-acetylaspartylglutamate (NAAG), scyllo-inositol, taurine, and guanine. Total NAA (tNAA: the sum of NAA and NAAG), total choline (tCho: the sum of GPC and PC), total creatine (tCr: the sum of Cr and PCr), and sum of Glu and Gln (Glx). To calculate the absolute metabolite concentrations, an unsuppressed water signal was used as a reference. Quantification estimates of 2HG were considered unreliable and were excluded when the Cramer-Rao lower bounds, returned as the percentage of standard deviation (%SD) by LCModel, was greater than 35%.

Sequencing for *IDH1* and *IDH2* was performed for four out of five cases with ample tissue for DNA analysis. DNA was extracted from fresh frozen tissue using the QIAamp Blood & Tissue Kit (Qiagen, Valencia, CA, USA) or using a formalin-fixed, paraffin-embedded (FFPE) tumor tissue using the QIAmap DNA FFPE Tissue Kit (Qiagen). PCR amplification was performed using the following primer sets: forward 5′-ACCAAGGATGCTGCAGAAGC-3′ and reverse 5′-AGATGGACGCCTATTTGTAAGT-3′ at codon 132 for the *IDH1* gene, and forward 5′-AGCCCATCATCTGCAAAAAC-3′ and reverse 5′-CAGTGGATCCCCTCTCCAC-3′ at codon 172 for the *IDH2* gene [3], which were different from those used in the original study [1].

For fluorescence in situ hybridization (FISH) for 1p/19q codeletion, formalin-fixed paraffin-embedded (FFPE) hematoxylin and eosin (HE)-stained tissue sections were examined to determine areas with high tumor cell density and a lack of necrosis and hemorrhage. Areas of interest on the HE-stained slides were transcribed to the reverse side of the unstained FFPE sections using a diamond-tipped marker. The FISH assay was performed on 4-µm-thick sections, using ZytoLight SPEC 1p36/1q25 and ZytoLight SPEC 19q13/19p13 probes for locus-specific 1p and 19q analysis, respectively, following the manufacturers’ instructions (ZytoVision, Bremerhaven, Germany). Additionally, 1p36 and 19q13 locus-specific red fluorescent probes were used as deletion targets and 1q25 and 19p13 green probes were used as internal controls. Nuclei were counterstained with 4,6-diamidino-2-phenylindole (DAPI). FISH was performed on a Z-stacked two-dimensional image using a fluorescence microscope BZ-9000 (KEYENCE, Osaka, Japan). A minimum of 100 adjacent, non-overlapping interphase nuclei were examined for each assay. Whereas normal diploid nuclei displayed a signal ratio of 2/2, a nucleus was considered to harbor a deletion if the target signal was 1 (i.e., 2/1) in relation to normal control signals. If the number of deleted nuclei was ≥50%, the tumor was considered to present a deletion of the targeted chromosome part [4].

## 3. Results

### 3.1. Rare IDH2 Mutation Detected in a Recurrent “False-Positive”Case

**Figure 1:**
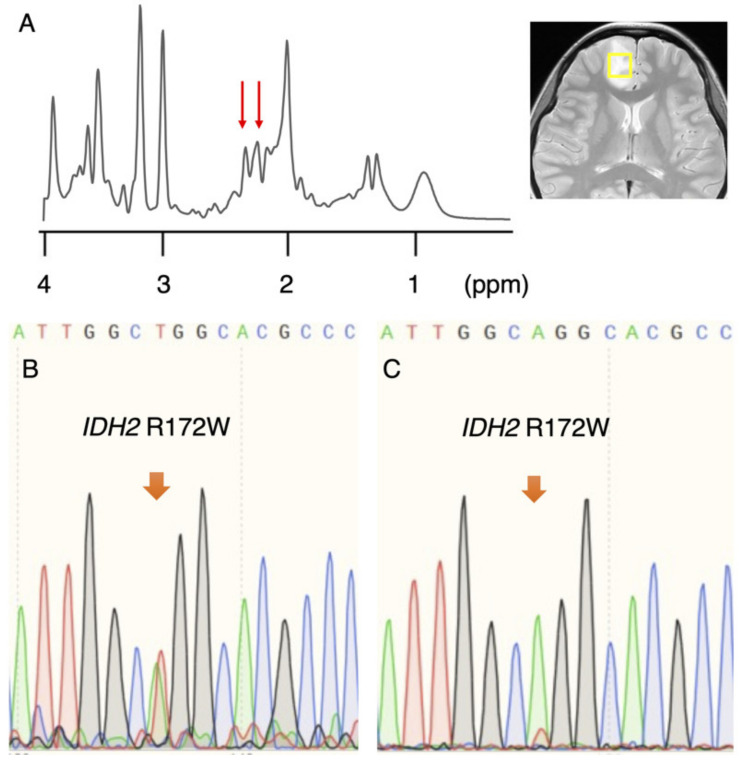
Rare *IDH2* mutation diagnosed at relapse. (**A**) Single voxel MRS (SVMRS) spectra showing small peaks (red arrows) at a chemical shift of approximately 2.25 ppm reflect the presence of 2-hydroxyglutarate (2HG). 2HG was quantified as 6.820 mM. (**B**) Obvious *IDH2* R172W mutation was detected at relapse. (**C**) Reassessment of *IDH2* mutation in the initial tumor revealed a small, mutant peak.

In the original study, a 2HG of 1.489 mM or higher was detected in 5 of 27 (18.5%) gliomas that were determined to be IDH-wildtype. Recently, the tumor was found to have recurred in one of the five patients, and subsequently, the patient underwent a second surgery. A single voxel MRS, taken before the first surgery, showed characteristic small peaks at a chemical shift of approximately 2.25 ppm, reflecting 2HG (Figure 1A) and a high 2HG accumulation of 6.820 mM. A molecular analysis of the recurrent tumor via Sanger sequencing using a different *IDH2* primer revealed an *IDH2* R172W mutation (Figure 1B), and the pathological diagnosis was anaplastic oligodendroglioma, IDH-mutant and 1p/19q-codeleted. We re-analyzed the *IDH2* status in the tumor from the initial surgery and found an *IDH2* R172W mutation, albeit with a very small mutant peak (Figure 1C).

### 3.2. Re-Evaluation of IDH1 and IDH2 Mutations in Remaining Cases

**Figure 2:**
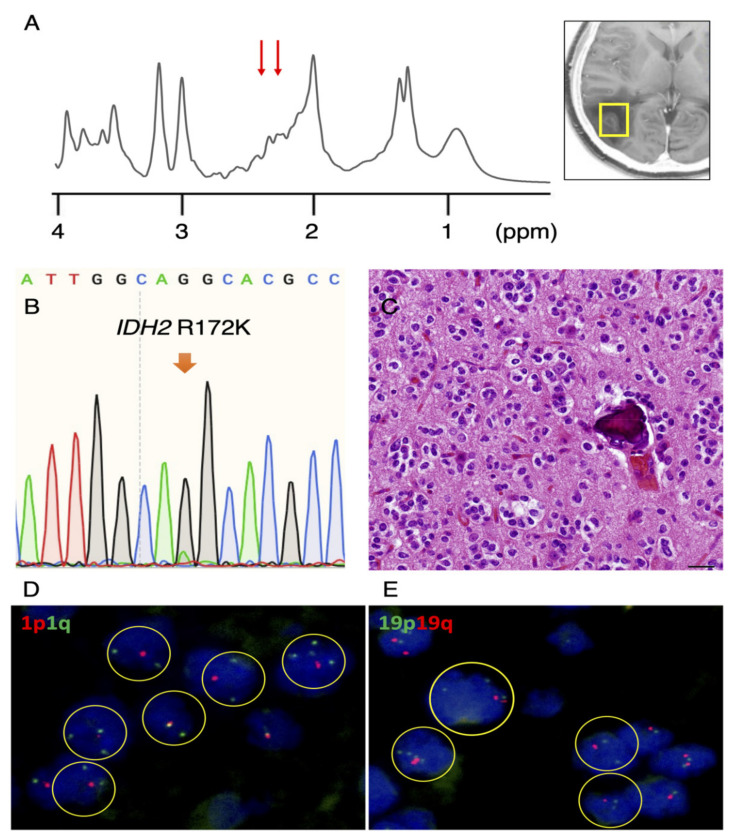
*IDH2* mutation identified in a second case. (**A**) SVMRS spectra show minimal peaks at 2.25 ppm (red arrows) and 2HG quantified to be 2.763 mM. (**B**) A small, IDH2 R172K-mutant peak was detected. (**C**) Morphologically, tumor cells with perinuclear halos lacking astrocytic processes, chicken-wire vessels and calcification were observed, suggestive of oligodendroglioma (scale bar = 20 μm). Codeletion of 1p (**D**) and 19q (**E**) were detected by fluorescence in situ hybridization (FISH).

Subsequently, we re-evaluated the *IDH1* and *IDH2* status in three out of the four remaining “false-positive” tumors with available tissue and found a *IDH2* R172K mutation in 1 patient with subtle peaks at a chemical shift of 2.25 ppm (Figure 2A). A very small mutant peak was detected using Sanger sequencing (Figure 2B). Morphologically, perinuclear halos, chicken-wire vessels and calcification were observed, all of which are characteristic of oligodendrogliomas (Figure 2C). Furthermore, 1p (Figure 2D) and 19q (Figure 2E) deletions were confirmed by fluorescence in situ hybridization. The tumor was diagnosed as an oligodendroglioma, IDH-mutant and 1p/19q-codeleted, according to WHO2016 [5].

Thus, we found that the MRS was correct, and that we initially failed to detect rare *IDH2* mutations in two out of the five cases. The specificity of 2HG, at a cutoff of 1.489 mM to determine IDH mutation, was calculated to be 81.3%, not 72.2%, as originally reported [1]. The demographics of the five cases are summarized below (Table 1).

We compared the metabolite concentrations of true-positive cases (*n* = 2) and false-positive cases (*n* = 3) (Table 2). Due to the small number of cases, the median concentration of metabolites between the two groups did not reach statistical significance. However interestingly, we found that the median GSH and Glu+Gln concentrations were lower, and the median Lac concentration was higher in the true-positive group than in the false-positive group, which is consistent with data that we [1,6] and others [7,8] have previously reported in IDH-mutant versus IDH-wildtype gliomas.

## 4. Discussion

In the present study, we re-evaluated five gliomas initially assessed to be of IDH wildtype but showed a high accumulation of 2HG and were thought to be false-positive. Two cases harbored rare *IDH2* R172 mutations that were not detected in the original analysis. The 2HG molecule contains five nonexchangeable protons, giving rise to multiplets at three locations on MRS: of approximately 4.02, 2.25, and 1.90 ppm [9]. The largest multiplet is located at 2.25 ppm. The detection of this multiplet is complicated by the spectral overlap of glutamate (Glu; 2.43 ppm), glutamine (Gln; 2.34 ppm), and gamma-aminobutyric acid (GABA; 2.28 ppm), all of which share the ^4^CH_2_ group [10]. This can be expected given the structural similarities of Glu, Gln and 2HG. The direct detection of the multiplet at 1.90 ppm is made difficult due to its proximity to the NAA resonance at 2.01 ppm, which shares ^3^CH_2_. Finally, the multiplet at 4.02 partially overlaps with creatine (Cr: 3.92 ppm), phosphocreatine (PCr; 3.94 ppm), myoinositol (Ins; 4.06 ppm), lactate (Lac; 4.1 ppm) and free choline (fCh; 4.05 ppm), sharing ^2^CH_2_ [9], which makes the unambiguous detection of 2HG challenging. A false-positive rate of approximately 22% was observed by Pope et al. using short-echo MRS (TE at 30 ms) for the detection of 2HG in gliomas [11]. This false-positive rate can be reduced by using long-echo MRS with TE at 97 ms and three-dimensional volume-localized basis (VLB) spectra, minimizing the effect of macromolecules in the detection of 2HG [9,12]. Other studies suggest that 2HG/[lipid + lactate] ratios [8] or a combination of 2HG and Glu [7] can provide a higher diagnostic accuracy than 2HG alone.

The sampling error must be considered when assessing the DNA of tumor tissues, especially when using frozen tissue. A recent study by Barritault et al. detected IDH1 and TERT promoter mutations from non-diagnostic biopsies from glioma patients using SNaPshot polymerase chain reaction [13], suggesting that methods that are more sensitive than Sanger sequencing may detect mutations in tissues with a low tumor volume. Since the mutant peak was small in both cases with detected *IDH2* mutation, we suspect that the tumor volume was low in these cases.

The limitations of this study include the small number of subjects, a suboptimal analysis of 2HG at a TE of 30 ms, the lack of ample tissue for DNA testing in one case, and an inability to detect rare IDH1/2 mutations differentiated from *IDH1* R132 and *IDH2* R172, such as *IDH1* R100Q. Thus, we were unable to determine the exact cause of the false-positive results in the remaining three cases.

One of the advantages of detecting 2HG by MRS is the non-invasive screening for rare mutations of *IDH1* and *IDH2*, as all IDH mutations are known to produce 2HG [14]. Highly reliable antibodies for IDH1 R132H, which constitutes approximately 90% of all IDH mutations in gliomas [3,15], are commercially available and widely used [16,17], but antibodies for other IDH1 and IDH2 mutant proteins are less widely available [18]. A recent, multicenter study suggested that non-IDH1-R132H IDH1/2 mutations are associated with an improved survival for astrocytomas compared to their R132H-mutant counterpart [19], which provides a basis for analyzing these rare mutations. In the present study, rare *IDH2* mutations were found in 2 out of 5 cases initially thought to be false-positive for 2HG. Both cases were pathologically diagnosed as oligodendrogliomas, which are known to almost uniformly harbor IDH mutations and 1p/19q codeletions [5].

## Figures and Tables

**Table 1:** Characteristics of the 5 presumed, false-positive cases.

Case No.	1	2	3	4	5
Age	30	51	59	72	67
Sex	F	F	M	F	F
Location	Rt Frontal	Rt Parietal	Rt Frontal	Rt Temporal	Lt Thalamus
2HG (mM)	6.820	2.763	5.589	4.477	5.448
Lactate (mM)	1.912	4.824	0.189	0.000	6.874
IDH1 R132H IHC	Negative	Negative	Negative	Negative	Negative
IDH 1/2 sequence	*IDH2* R172W	*IDH2* R172K	WT ^6^	WT ^6^	N/A ^4^
Mutant peak	Small	Small	None	None	N/A ^4^
1p/19q FISH	1p/19q codel ^1^	1p/19q codel ^1^	N/A ^4^	N/A ^4^	N/A ^4^
Pathological diagnosis	OD ^5^	OD ^5^	DA ^2^	GBM ^3^	DA ^2^

^1^ codeleted; ^2^ diffuse astrocytoma; ^3^ glioblastoma; ^4^ not available/not applicable; ^5^ oligodendroglioma; ^6^ wildtype.

**Table 2:** Comparison of metabolites between true-positive and false-positive cases.

Metabolite	Median Concentration (mM) True-Positive Cases (*n* = 2)	Median Concentration (mM) False Positive Cases (*n* = 3)	*p*-Value
GSH ^1^	1.020	1.839	0.40
2HG ^2^	4.792	5.448	>0.99
mI ^3^	5.561	3.862	0.40
Lac ^4^	4.393	0.189	0.40
tCh ^5^	1.563	1.860	0.80
tNAA ^6^	3.165	4.085	0.40
tCr ^7^	3.803	5.890	0.80
Glu+Gln ^8^	5.055	12.35	0.20

^1^ glutathione; ^2^ 2-hydroxyglutarate; ^3^ myoinositol; ^4^ lactate; ^5^ total choline; ^6^ total N-acetylaspartate; ^7^ total creatine; ^8^ glutamate + glutamine.

## Data Availability

The datasets analyzed during the current study are available from the corresponding author upon request.
